# Novel Molecules in Diabetes Mellitus, Dyslipidemia and Cardiovascular Disease 2.0

**DOI:** 10.3390/ijms25179527

**Published:** 2024-09-02

**Authors:** Cosmin Mihai Vesa, Simona Gabriela Bungău

**Affiliations:** 1 Department of Preclinical Disciplines, Faculty of Medicine and Pharmacy, University of Oradea, 410073 Oradea, Romania; cosmin.vesa@csud.uoradea.ro; 2 Doctoral School of Biomedical Sciences, University of Oradea, 410087 Oradea, Romania; 3 Department of Pharmacy, Faculty of Medicine and Pharmacy, University of Oradea, 410028 Oradea, Romania

Diabetes mellitus, dyslipidemia and cardiovascular disorders represent very prevalent chronic diseases in developed countries contributing to a high morbidity and loss of quality of life [1]. New medication recently introduced in clinical practice such as SGLT2 inhibitors [2], GLP-1 agonists [3], GIP agonists [4] and PCSK9 inhibitors [5] have demonstrated their efficacy in large clinical trials. However, continuous research represented by experimental studies performed in vitro or in vivo can offer new perspectives mainly regarding their pleiotropic effect. This was the case with SGLT-2 inhibitors and GLP-1 agonists, initially developed for type 2 diabetes treatment and for their glucose-lowering effect; these drugs demonstrated blood pressure reduction, weight reduction, lipid profile improvement, anti-atherosclerotic effects, improvement in cardiac function and renal protection [6]. Nonetheless, repurposing old drugs such as metformin [7] or statins [8] for addressing newly discovered physio pathological pathways in these disorders is of much interest. Not only is the treatment important but also novel molecules can serve as important laboratory diagnostic markers and can predict the apparition of complications of diabetes mellitus.

Novel molecules in the treatment and diagnosis of diabetes mellitus, dyslipidemia and cardiovascular disorders is a continuing developing chapter in molecular sciences, where these molecules are used both as diagnostic tools (biochemical markers and miRNAs) and as a therapeutical means for addressing the above-mentioned disorders. Continuous research in the field leads to the personalization of cardio-metabolic treatment and better outcomes for the patients.

The summary below presents the most important results obtained by the researchers who published in our Special Issue.

Bakhashab S. et al. demonstrated that miR-199b-5p expression in colony-forming units–Hill’s colonies obtained from type 1 diabetes mellitus patients was reduced after metformin administration, with miR-199b-5p known to be a proatherogenic molecule (Contribution 1). Dłuski D.F. showed that circular RNA hsa_circ_0002268 is overexpressed in pregnant women from Poland suffering from gestation diabetes and that it could serve as a diagnostic marker (Contribution 2).

Another important research conducted by Höpfinger A. et al. revealed that in murine and human models, cathelicidin antimicrobial peptides play complex roles in mediating the interaction between inflammation and cardiovascular disease (Contribution 3). The review written by Dalle S. discussed the potential role of targeting 12 protein kinases such as tumor progression locus 2 kinase (TPL2) or inhibitor of nuclear factor kappa-B kinase subunit beta (IKKβ) for improving beta cell function and preventing diabetes mellitus (Contribution 4). The manuscript of Chatzianagnostou, K et al. presented the results obtained in clinical trials as well as in experimental studies of GLP-1 agonists and SGLT-2 inhibitors demonstrating novel potential applications of these drugs—such as producing epigenetic modifications or reducing oxidative stress (Contribution 5).

Sirca T.B. et al. demonstrated that polyphenol administration can influence paraoxonase 1 activity, an enzyme involved in protection against diabetes mellitus, nonalcoholic fatty liver disease and many other metabolic diseases (Contribution 6). In an extensive review, Has I.M. discussed the interaction between food polyphenols and gut microbiota, concluding that a high fruits and vegetables diet can increase the prevalence of gut bacteria, having anti-inflammatory and antioxidant effects, which results in improving the health of metabolic diseases patients (Contribution 7).

Despite many advances, the field of cardio-metabolic diseases remains a challenge due to the high prevalence of complications and mortality. Continued research, both in vitro and in vivo, is needed to discover new molecules involved in pathogenesis, diagnosis, prognosis and treatment. Moreover, the promotion of molecular research in diabetes, dyslipidemia and cardiovascular diseases to find new pathophysiological pathways and potential treatments still offers vast potential for exploration.
